# Items

**Published:** 1903-01

**Authors:** 


### ITEMS.

THE *Annals of Surgery* for December completes the eighteenth year of its publication. In the fulness of its table of contents, and in the important character of its articles, it quite equals, if it does not excel, any number that has preceded it; and this is saying much, since from the first number this magazine has been the medium through which the best surgical writers and teachers on both sides of the Atlantic have published their work. The table of contents includes articles on genitourinary surgery, anatomy of the pelvis and abdomen, rupture of the kidney, and several papers on abdominal surgery besides many short abstracts. It should be examined to be appreciated.

THE Physicians' Daily Memorandum for 1903, published by the M. J. Breitenbach Company, of New York, makes its appearance with promptitude. It is one of the most useful of desk reminders, being issued in the same style as in former years, and can be had by addressing the publishers, 53 Warren Street.

THE Antikamnia Calendar for 1903 is another artistic water color by Helen Hyde and is entitled, "His First Temple Offering," (the Joss stick). On the reverse is a Calendar with some useful antikamnia notes. Duplicates can be had by sending 10 cents to the Antikamnia Company, St. Louis, Mo.

THE Daily Reminder Calendar (1903) of Scott and Bowne is a handy equipment for the desk, one slip for each day in the year, each one having printed upon it a note concerning Scott's emulsion. The publishers will send it upon application. Address, 411 Pearl Street, New York.

THE New York Pharmacal Association, Yonkers, N.Y., present to the profession an artistic folder calendar, containing portraits of eminent physicians, of ancient and modern times. The calendar embraces the period between October 1, 1902, to October 1, 1903.

THE Denver Chemical Manufacturing Company is sending their "Antiphlogistine" Christmas folder to every physician in America and England. It is a calendar and a reminder of the uses of antiphlogistine as well; and besides it is an artistic little souvenir of the season.

